# Keystone actors do not act alone: A business ecosystem perspective on sustainability in the global clothing industry

**DOI:** 10.1371/journal.pone.0241453

**Published:** 2020-10-30

**Authors:** Jacob Hileman, Ivan Kallstenius, Tiina Häyhä, Celinda Palm, Sarah Cornell

**Affiliations:** Stockholm Resilience Centre, Stockholm University, Stockholm, Sweden; University of York, UNITED KINGDOM

## Abstract

Global industries are typically dominated by a few disproportionately large and influential transnational corporations, or keystone actors. While concentration of economic production is not a new phenomenon, in an increasingly interconnected and globalized world, the scale of the impacts of keystone actors on diverse social-ecological systems continues to grow. In this article, we investigate how keystone actors in the global clothing industry engage in collaboration with a variety of other organizations to address nine interrelated biophysical and socioeconomic sustainability challenges. We expand on previous theoretical and empirical research by focusing on the larger business ecosystem in which keystone actors are embedded, and use network analysis to assess the contributions of different actor types to the architecture of the ecosystem. This systemic approach to the study of keystone actors and sustainability challenges highlights an important source of influence largely not addressed in previous research: the presence of organizations that occupy strategic positions around keystone actors. Such knowledge can help identify governance strategies for advancing industry-wide transformation towards sustainability.

## Introduction

The majority of all global industries, both old and new alike, are dominated by a few disproportionately large transnational corporations [1,2]. This phenomenon has been observed across such diverse industries as fisheries [3,4], genetic resources [5,6], food security [7], cement and fossil fuel production [8], agricultural seed supplies [9], and international finance [10,11]. The asymmetrical distribution of influence in global industries arises as a result of market consolidation and the corporate power that comes with claims on large market shares [12]. In the case of resource extraction and provision, this phenomenon has been described as a “keystone pattern” [13], and the large transnational corporations driving this phenomenon may be considered “keystone actors” in global social-ecological systems [4].

Concentration of economic production is not a new phenomenon, yet in an increasingly interconnected and globalized world, the scale of the impacts that keystone actors have on diverse social-ecological systems is greater than ever before [1]. As such, keystone actors are an important study object in the interdisciplinary fields of environmental governance and sustainability science. While a rich body of empirical literature continues to develop around the study of keystone actors in complex social-ecological systems [e.g., 3–5], keystone actors are not islands of corporate influence. There is a growing need to understand how the biophysical and socioeconomic footprint of keystone actors is in turn influenced by the “business ecosystem”–the network of relationships among companies, non-governmental organizations (NGOs), trade associations, multi-stakeholder platforms, and other organizations—in which keystone actors are embedded [14,15]. Achieving industry-wide transformation towards sustainability requires coordinated action among the multitude of actors that comprise the ecosystem. This will not occur if keystone actors are studied in isolation.

In this article, we focus on the global clothing industry—one of the largest industries in the world, and with far-ranging social-ecological impacts [16]—and assess how keystone actors collaborate with a range of other organizations to address nine interrelated biophysical and socioeconomic sustainability challenges. Drawing on research from the environmental governance literature, we use network analysis to assess the structure of the business ecosystem in which global efforts to transform unsustainable practices in the clothing industry are taking place, and to illuminate relational structures that enable or constrain collaboration among actors in the ecosystem. We are further able to pinpoint the location of keystone actors within the business ecosystem, as well as identify other actors who may be in position to influence keystone actors and other actions in the ecosystem. Such information is necessary for the development of governance strategies for transforming global industries.

This article advances the study of keystone actors in global social-ecological systems through connecting with parallel research in the business ecosystem literature [e.g., 15, 17–20]. Merging these two research streams also has the added benefit of helping to address important knowledge gaps in each. Within the social-ecological systems literature, research on keystone actors has primarily focused on the impacts they have on different planetary boundaries and the biosphere, and only recently started to consider the complex networks in which keystone actors are embedded [3,11]. Conversely, within the business ecosystem literature, research on “keystones” has focused on the wider networks of influence these actors affect, and are affected by, and has largely not considered their biophysical and socioeconomic impacts [21]. Using a network-based analytical approach, this article builds on ideas posited in recent research regarding connectivity and keystone actors in global industries [3,4,22], and serves to operationalize the language of “networks” that is typically used to describe business ecosystems. Lastly, this article provides a generalizable and straightforward method for studying keystone actors within business ecosystems, which can broadly be applied to any global industry.

### Aligning research on keystone actors and business ecosystems

The term “keystone actor” is derived from the concept of “keystone species” in systems ecology [23,24], which refers to those species in an ecosystem having outsized influence in spite of being relatively few in number [25]. The analogy to transnational corporations is imperfect, as the ways in which companies and other organizations interact in an industry are different than biological organisms in a natural ecosystem. Recognizing these differences, Österblom et al. [2015, p.11] posited four criteria that refine when large transnational corporations fill the role of keystone actors: “a) [keystone actors] dominate global production revenues and volumes within a particular sector, b) control globally relevant segments of production, c) connect ecosystems globally through subsidiaries and d) influence global governance processes and institutions.” They used these criteria to illustrate how thirteen companies control up to 40% of some the most valuable fishing stocks, and can be considered keystone actors in global fisheries.

In spite of the growing number of studies addressing the keystone pattern in global industries, it is presently challenging to identify and synthesize individual studies of keystone actors due to considerable variation in the terminology used to describe the phenomenon. For example, in the global financial sector these actors have been termed “financial giants” [11] and “super-entities” [2]. The language of keystone actors is, however, already being used in a number of parallel literature streams, including the business ecosystem literature [14,26,27]. Yet the term is far from being used in a congruent fashion. For example, Iansiti and Levien [14] describe how “keystones” provide stability within dynamic business ecosystems, and do so at least in part to protect their own growth and capital. Empirical knowledge has been slow to accumulate across individual studies of keystone actors on account of these discrepancies.

The business ecosystem concept is a useful construct for understanding the influence keystone actors wield [21], and conversely, how keystone actors may be influenced through their interactions with other actors. Though lacking a concise definition, the term “business ecosystem” generally describes the network of interactions that arises between companies and other organizations as they cooperate and compete to deliver on their value proposition [14,15,20,26]. Depending on the specific value proposition, a business ecosystem may consist of a single industry, or span multiple industries. There is no formula for defining the boundaries of a specific business ecosystem; it must be empirically defined. In our consideration of the global clothing industry, we do not focus on the items of clothing themselves, but rather efforts to address sustainability challenges in the production, distribution, and consumption of clothing.

### Integration through a network-based analytical approach

In this article, we merge research on keystone actors and business ecosystems through the use of network analysis. While studies of keystone actors and business ecosystems draw heavily on the language of networks [14,15,28–30], few empirical investigations in the broad social-ecological systems literature have addressed these topics using a network-based approach [e.g., 22]. The use of network analysis is, however, rapidly growing in studies of environmental governance across a range of empirical contexts, and from the local to the global levels [e.g., 31–36]. Many of the general research questions are also similar across these literature streams.

Research on environmental governance networks broadly centers on the relationship between structure and function in governance arrangements [37–40]. Most empirical studies within this field of research investigate how specific structures of collaboration among diverse public and private actors may facilitate, or hinder, governance processes associated with improved social-ecological outcomes [e.g., 32, 39, 41–45]. This research framing is equally applicable to the study of business ecosystems, when the intention is to understand how structures of collaboration among actors facilitate, or hinder, coordination of industry-wide goals and related processes. The focus of this study is primarily on closed and open network structures. Closed structures (i.e., densely interconnected groups, or communities, of actors) help facilitate cooperation to address systemic problems that cannot be resolved by any one individual or organization [38,46]. Conversely, open structures (i.e., highly central actors possessing substantially more ties than other actors in the system) help facilitate coordination around information diffusion and project implementation [37,38]. Most networks possess both these structures to varying degrees [47–49]. Actors that contribute to these structures may be important leverage points in business ecosystems, on account of their ability to facilitate different forms of multi-actor collaboration.

### Social-ecological impacts of the global clothing industry

With an estimated worth of $2.4 trillion USD, and employing tens of millions of workers worldwide, the global clothing industry constitutes one of the largest industries in the world [50,51]. Coupled with long global supply chains—beginning with the production of virgin materials (e.g., cotton, polyester) and moving on through the manufacturing, distribution, and post-consumer journey that every clothing item takes—the clothing industry provides a rich empirical context for examining keystone actors and sustainability challenges. Recent estimates suggest around 150 billion items of clothing were produced in 2017, and that by 2030 production is projected to rise by 63% in order to meet the demand for clothing from a growing global middle-class population [52,53]. Even at current production levels, the industry is estimated to generate over 90 million metric tons of solid waste annually [54]. One report recently found the clothing industry is already operating beyond all the planetary boundaries [52], with the exception of fresh water consumption [55]. The industry has also come under scrutiny in a number of countries for low wages, long working hours, and lapses in safe working conditions, including the 2013 collapse of a garment factory in Bangladesh that claimed over 1,000 workers’ lives [56,57].

Actions are currently being taken at the global level to address the biophysical and socioeconomic impacts of the clothing industry. Internally, clothing companies produce corporate sustainability reports that define goals and targets, and provide a roadmap of where a company is heading in its efforts to tackle sustainability within its supply chain. However, companies often do not disclose the data they use to determine how they are performing with respect to their targets [58]. In response, there is increasing pressure from policymakers and consumers for clothing companies to be transparent, and to adopt trackable industry reporting metrics that capture aspects of biosphere stewardship, circular economy, and planetary boundaries [59]. Focusing only on the internal operations of fashion textile companies, however, fails to recognize the potential of other types of actors to influence the direction of global sustainability efforts within the industry, which is operationalized through wide-ranging industry collaborations. Global sustainability efforts involve a variety of other organizations (e.g., trade associations, non-governmental organizations, multi-stakeholder platforms), and collaboration is increasingly being recognized as a critical component of industry efforts to address sustainability challenges.

## Materials and methods

The data for this study come from corporate sustainability reports and other publicly available sources of information on actors’ websites. The dataset consists of the array of keystone actors and other organizations working on sustainability challenges in the clothing industry at a global level; the biophysical and socioeconomic sustainability challenges each actor addresses; and the collaboration occurring among actors to address these sustainability challenges. Given our interest in understanding how the business ecosystem is structured at present, and in highlighting the value of network analysis as a tool for merging the study of keystone actors and business ecosystems, we focus on a suite of descriptive network statistics. We examine network density and average local clustering coefficient as measures of closed structures; average path length, degree centralization, and median degree as measures of open structures; and the external-internal (E-I) index as a measure of which types of actors collaborate significantly more, or less, frequently in the business ecosystem. To capture how the set of sustainability challenges is distributed throughout the ecosystem, we assess the variation in the challenges each actor type engages with.

### Identification of keystone actors

**Fig 1:**
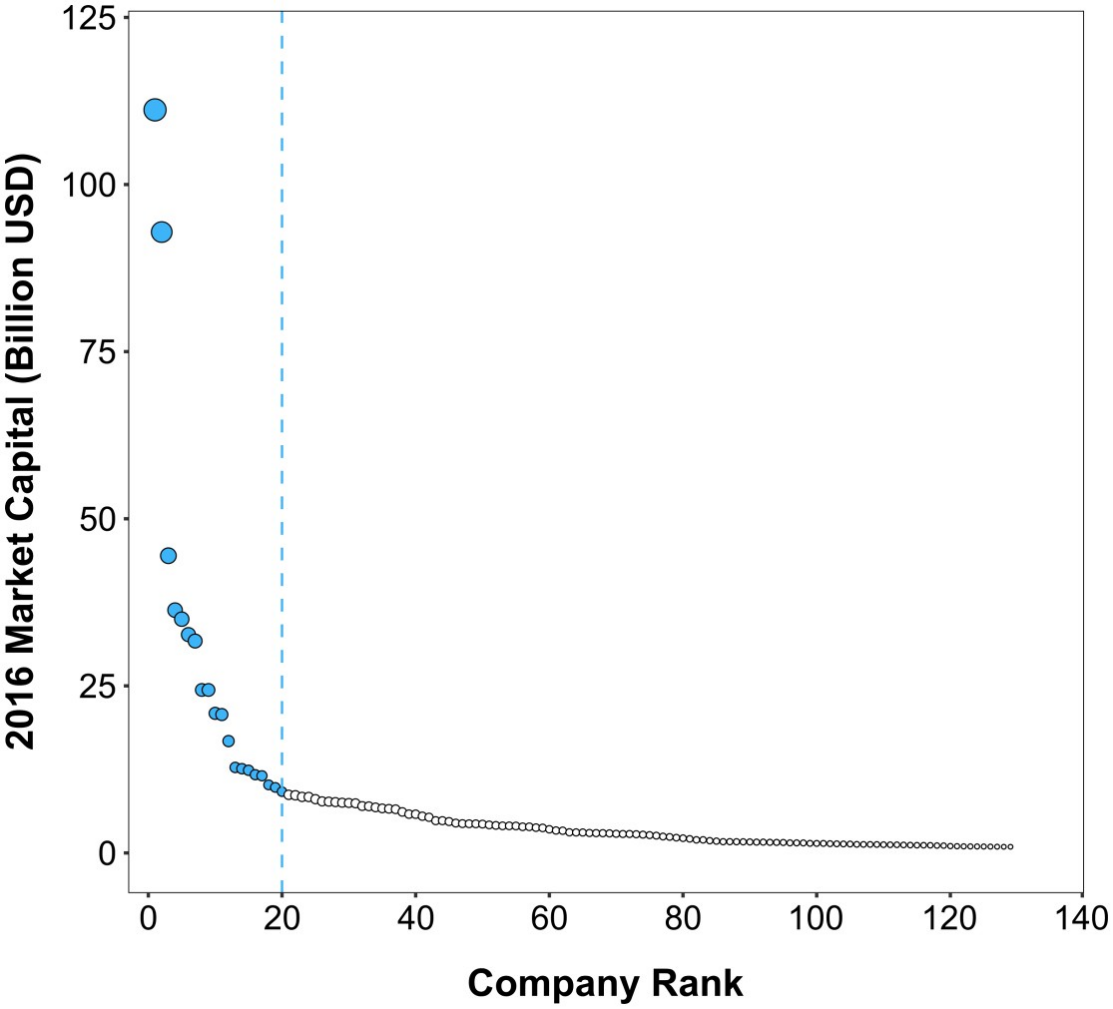
Keystone pattern in the global clothing industry. Data points are sized by market capital, and the blue points and dashed line indicate the population of keystone actors in this study.

We used Fashion United’s list of the top 200 fashion companies to identify the suite of keystone actors in the global clothing industry [60]. We began by removing all companies from the list that were focused on fashion and luxury items other than textile-based clothing (e.g., jewelry, watches, perfume, leather goods), after which 129 clothing companies remained. While each of these companies is large in its own right, individually possessing over $900 million USD in market capital, there is nevertheless a discernable keystone pattern [13] when the companies are plotted by market capital (Fig 1).

There is no standard methodology for determining the cutoff point that separates keystone actors from all other companies in a given industry. While there appears to be a critical point around $17 billion USD among clothing companies, market capital is a relatively dynamic metric. Here, we define the population of keystone actors as those clothing companies with more than $9 billion USD in market capital, which corresponds to the top twenty clothing companies on the Fashion United list. Erring on the side of a potentially lower threshold for keystone actors in the industry makes it less likely that we might accidentally exclude a keystone actor from the analysis, which in turn reduces the likelihood that we might fail to identify other important organizations in the business ecosystem during the subsequent network data collection process.

Among large clothing companies, these twenty corporations stand out by collectively possessing 62% of the total market capital held by the top 129 clothing companies. McKinzey & Co. [51] also found the top twenty fashion companies reap 97% of profits in the industry. These findings satisfy the first criterion of keystone actors. While companies do not typically make detailed data about their supply chains publicly available, the general information they do provide regarding the locations of their suppliers is sufficient to satisfy the second and third criteria of keystone actors. This is further reinforced by the Open Apparel Registry’s database of over 60,000 suppliers, which provides a rich picture of the vast global reach of the clothing industry [61]. Lastly, as the findings will later demonstrate, most of these companies have considerable presence in global platforms and other governance mechanisms, and satisfy the fourth criterion of keystone actors.

### Building the business ecosystem network

A network describes any relational system composed of a set of interconnected nodes and links. In this study, nodes are defined as organizational actors (e.g., clothing companies, NGOs, multi-stakeholder platforms). Links between actors represent collaboration to address sustainability challenges in the global clothing industry, not material flows or other forms of supply chain mapping. Furthermore, links represent inter-organizational collaboration, not intra-organizational collaboration occurring among the many complex hierarchical units within a company or brand.

To begin the data collection process, we reviewed the most recent corporate sustainability report for each of the identified keystone actors in order to determine who they work with to address sustainability challenges in the industry. Not all of the keystone actors produce sustainability reports annually, hence at the time of data collection the reports we reviewed were from 2016 or 2017. Furthermore, four of the keystone actors either did not produce publicly accessible sustainability reports at that time, or did not identify any organizations they collaborate with to address sustainability in their supply chains. Tellingly, these four clothing companies were not named as collaborators by any other actor in subsequent rounds of the data collection process, which suggests they are largely absent from the business ecosystem under consideration here.

**Fig 2:**
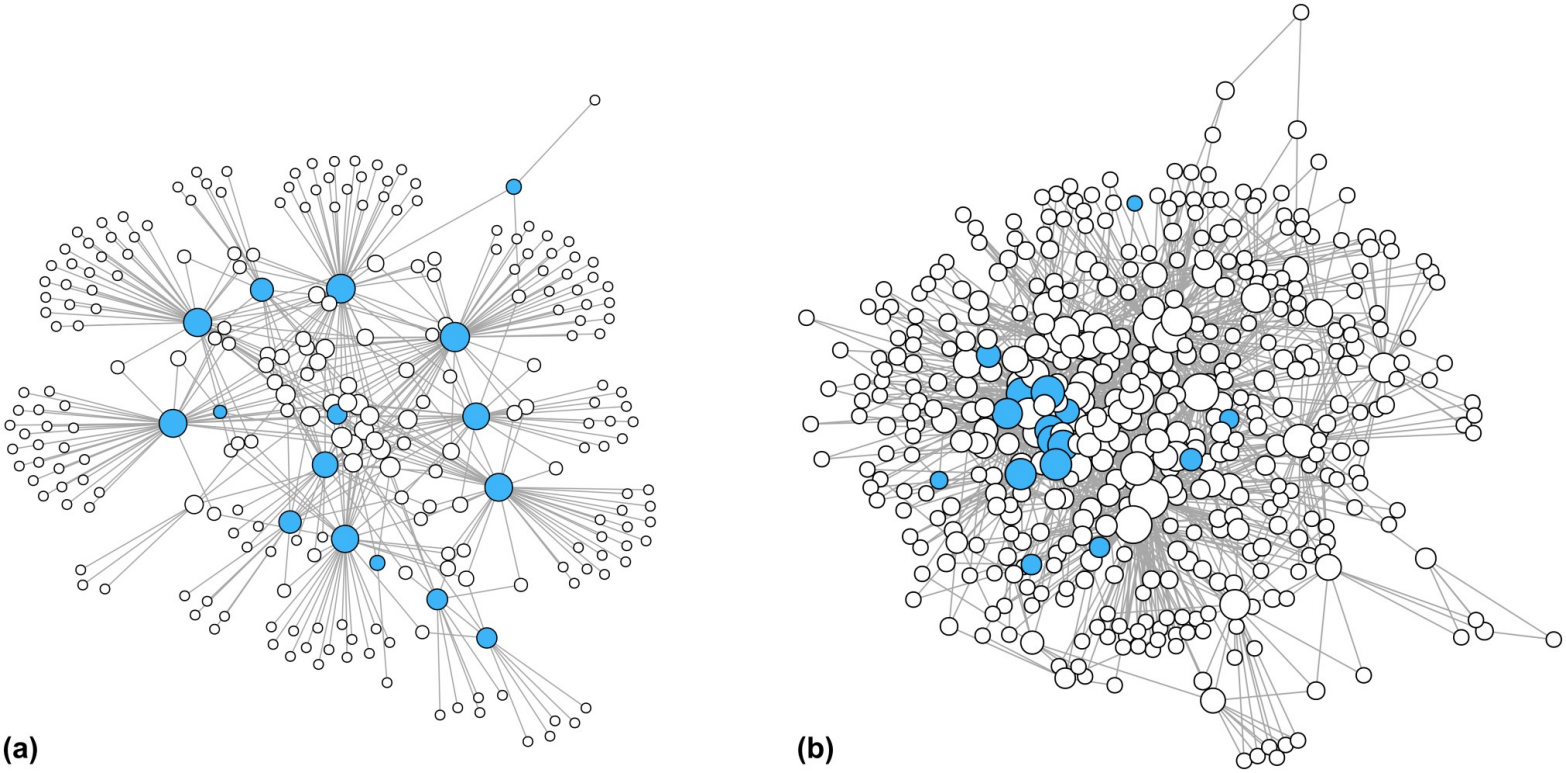
Network of keystone actors and partner organizations, and the full business ecosystem. a) Network of keystone actors (blue nodes) and other organizations from the first round of data collection. b) Location of keystone actors within the full business ecosystem. In each case, node size reflects the number of partners each actor possesses.

For the sixteen keystone actors that did produce sustainability reports, we reviewed the reports and recorded all the organizations each company named as partners. The resulting network of collaboration (Fig 2a) showcases how each keystone actor has its own set of unique partners, or niche, in the network, and highlights the presence of a core group of organizations in the center of the network that are connected to multiple keystone actors. Next, we allowed the data collection to snowball through reviewing publicly available information found on the websites of the identified partner organizations. We subsequently recorded the partners for all these organizations that satisfied the following two criteria: 1) they work at the global level (i.e., their scope of operations spans multiple world regions); and 2) their work specifically addresses sustainability challenges within the clothing industry. These criteria represent the boundaries of the network, i.e., which actors are included, and which are excluded. Without boundaries, the network under consideration here would potentially include every single actor in the global clothing industry, and present an impossible data collection task. We also declined to review any additional clothing brands or companies, due to both the challenge of data collection and the expectation that keystone actors will be engaged with the largest and most globally influential organizations in the industry.

For the actors satisfying the network boundary conditions, we reviewed their websites and recorded all the organizations they listed as partners in their efforts to address sustainability in the clothing industry. We concluded the snowball sampling after three rounds of data collection, at which point we identified no new organizations that satisfied the network boundary criteria. In all, we identified 1,938 unique actors. However, we excluded all actors with only one partner, in order to focus on the core structures at the heart of the business ecosystem network. Previous research has demonstrated core network structures tend not to vary substantially across different data collection methods [62], and preserve the dominant features of the overall network [47]. After removing these actors, the business ecosystem comprises 455 actors and 1,455 collaborative ties (Fig 2b).

Next, we recorded information about the attributes of the actors in the business ecosystem. We first classified actors into ten types of organization (Table 1): keystone actors, other clothing brands and companies, retailers, manufacturers, trade associations, multi-stakeholder platforms, NGOs, scientific research organizations, government institutions, and an “other” group comprising actors lacking an express focus on clothing (e.g., consulting firms, foundations, and companies not focused on clothing or other textile products). The actor typology generally reflects the role of each actor in the business ecosystem.

**Table 1:** Coding scheme for each actor type in the business ecosystem.

Actor Type	Description
Keystone actor	Any clothing company with more than $9 billion USD in market capital, according to the Fashion United list of the top 200 fashion companies [60].
Clothing brand/ company	Clothing brands/companies with less than $9 billion USD in market capital.
Retailer	A company that sells other companies’ clothes (retailers may also have their own brands).
Manufacturer	A producer of textile fibers and/or garments.
Trade association	A membership-based organization formed around a particular sector within the clothing industry (e.g., sportswear)
Multi-stakeholder platform	An initiative, program, project, or similar membership-based organization that invites broad participation from multiple interests and/or sectors within the clothing industry.
NGO	Non-profit and similar organizations dealing with issue advocacy and/or project development and implementation within the clothing industry.
Scientific research	Private or public laboratories, firms, or institutes focused on basic scientific research for developing new technologies and other innovations in the clothing industry.
Government institution	Formal government entities, such as ministries or inter-governmental institutions, having regulatory authority within their geographic and administrative jurisdictions.
Other	Organizations lacking an express focus on clothing that do not fit into the categories above, such as consulting firms, foundations, and other private enterprises.

Finally, we coded the actors according to nine sustainability challenges (Table 2): hazardous chemicals, waste reduction, energy consumption, air emissions, land use, recycling, wastewater, water conservation, and socioeconomic wellbeing (e.g., labor rights, working conditions). We arrived at this set of challenges through reviewing the corporate sustainability reports from the set of keystone actors, and classifying the different aspects of sustainability they collectively reported on. We do not consider more indirect actions, such as responsible sourcing practices and philanthropy, in order to focus on the sustainability challenges keystone actors are able to address directly. Each sustainability challenge is a binary variable, and we recorded ones and zeroes, respectively, to denote the sustainability challenges each actor does, and does not, address. It was not sufficient for an actor to acknowledge a particular sustainability challenge; there had to be clear indication that the actor was working to address the challenge.

**Table 2:** Coding scheme for biophysical and socioeconomic sustainability challenges.

Sustainability Challenge	Description
Hazardous chemicals	Actions to phase out, minimize the use of, and/or seek non-toxic replacements for hazardous chemicals used to manufacture clothes.
Waste reduction	Actions to minimize raw material inputs, minimize solid waste outputs, and/or improve the efficiency of industrial processes generating waste.
Energy consumption	Actions to minimize energy inputs, improve the efficiency of energy-intensive industrial processes, and/or use renewable energy sources throughout the supply chain.
Air emissions	Actions to reduce and/or recover airborne pollutants generate through the manufacturing of clothes (CO_2_ emissions are included under “energy consumption”).
Land use	Actions to minimize impacts on terrestrial ecosystems, such as on-farm best management practices for cotton, or reforestation/conservation programs for tensile.
Recycling	Actions to repurpose post-consumer garments through collection and direct reuse and/or recycling into raw fibers. This category also accounts for actions taken to use recycled cotton, polyester, and other materials for new fiber and garment production.
Wastewater	Actions to reduce the quantity and/or improve the quality of effluent from industrial processes (e.g., dyeing, washing) during the manufacturing of clothes.
Water conservation	Actions to minimize the water required and/or improve the efficiency of water used in the manufacture of clothing.
Socioeconomic wellbeing	Actions to prevent discrimination, child labor, provide living wages and safe working conditions, and otherwise protect the rights of those employed in the clothing industry.

### Network analysis methods

We analyze the business ecosystem as an undirected network of collaborative ties between actors, and assess both system- and group-level network structures to capture different aspects of collaboration. Using the “igraph” package [63] in the R Environment for Statistical Computing [64], we measure the following system-level network structures: density, average local clustering, average path length, median degree, and degree centralization.

Density and average local clustering coefficient are system-level measures related to network closure. Density captures connectivity in a network and represents the fraction of the total possible ties that are present among actors. The average local clustering coefficient is an indicator of the presence of dense subgroups, or communities of actors, in a network. The measure is obtained by first calculating the fraction of each actor’s partners that are themselves interconnected, and then calculating the average value across all actors in the network.

Degree centralization, median degree, and average path length are system-level measures related to open network structures. Degree centralization is an indicator of the extent to which a network is dominated by one or more high-degree actors (i.e., actors with substantially more ties than all others in the network). Median degree is a measure of connectivity in a network, and represents the median number of relationships per actor in a network. Average path length is a measure of relational proximity, and represents the mean number of ties that constitute the shortest path between any two actors in a network.

We use UCINET [65] to calculate the E-I index at the group level in the business ecosystem. The E-I index is a square matrix representing, in this case, the propensity of each different type of actor to collaborate, or not collaborate, with each other type of actor in the network. Collaboration between different types of actors is considered “external,” while collaboration among the same type of actor is considered “internal.” The E-I index helps to uncover whether certain forms of inter-organizational collaboration are more prevalent than others in the business ecosystem. We also assess median degree and average local clustering coefficient at the group level, which is accomplished by calculating the two measures within each actor type, as opposed to calculating the measures across all actors. Group-level network structures provide an indication of how each different type of actor in the business ecosystem contributes to the overall structure of the network.

## Results

System-level network structures provide an overview of the business ecosystem in which global efforts to transform unsustainable practices in the clothing industry are taking place. The density score indicates collaboration is relatively sparse in the business ecosystem, as only 1.4% of the total possible ties are present among the actors. This low value is not unexpected, given the large number of actors in the ecosystem, and should not be interpreted as suggesting little overall collaboration is taking place to address sustainability challenges. The same is true of the median degree (3), which indicates the majority of actors collaborate with three or fewer other actors; however, a small number of actors collaborate with a large number of others. The average local clustering coefficient (0.22) indicates the presence of communities of actors in the business ecosystem, but that the ecosystem overall is not characterized by closed structures occurring among most actors. The average path length (2.9) indicates relatively close proximity between actors in the business ecosystem—in a relational sense, not geographically—as actors tend to have three degrees of separation between all others actors. The centralization score (0.32) indicates the presence of one or more actors that are engaged in substantially more collaborative relationships than others. These results suggest the business ecosystem is characterized by predominantly open network structures.

Group-level network structures highlight the unique contributions of different actor types to the architecture of the business ecosystem (Table 3). The median degree of keystone actors (26) indicates they engage in considerably more collaborations than all other actor types. Keystone actors also have the lowest average local clustering coefficient (0.08), demonstrating that they tend to contribute more open structures to the business ecosystem. The median degree of multi-stakeholder platforms (6) indicates they are the second most well-connected actor type in the business ecosystem. They also have a relatively low clustering coefficient (0.14) compared to other actor types, indicating they similarly contribute to the open structure of the overall ecosystem. All other types of actor have a median degree of two or three, and considerably higher clustering scores. Scientific research organizations and trade associations have the highest clustering scores, and are the main drivers of closed structures in the business ecosystem. While it is more difficult for actors with many ties to have higher clustering scores, the measure is nevertheless an important indicator of the ways in which actors engage in collaboration to address sustainability challenges in the global clothing industry.

**Table 3:** Group-level descriptive statistics for each actor type.

Actor Type	Actor Count	Median Sustainability Challenges	Median Degree	Average Local Clustering Coefficient
Keystone actor	16	8	26	0.08
Clothing brand/company	134	7	2	0.21
Retailer	37	7	2	0.15
Manufacturer	31	6	2	0.20
Trade association	31	0	2	0.42
Multi-stakeholder platform	66	1	6	0.14
NGO	28	2	3	0.22
Scientific research	17	5	2	0.41
Government institution	6	1	3	0.25
Other	89	4	2	0.26

**Fig 3:**
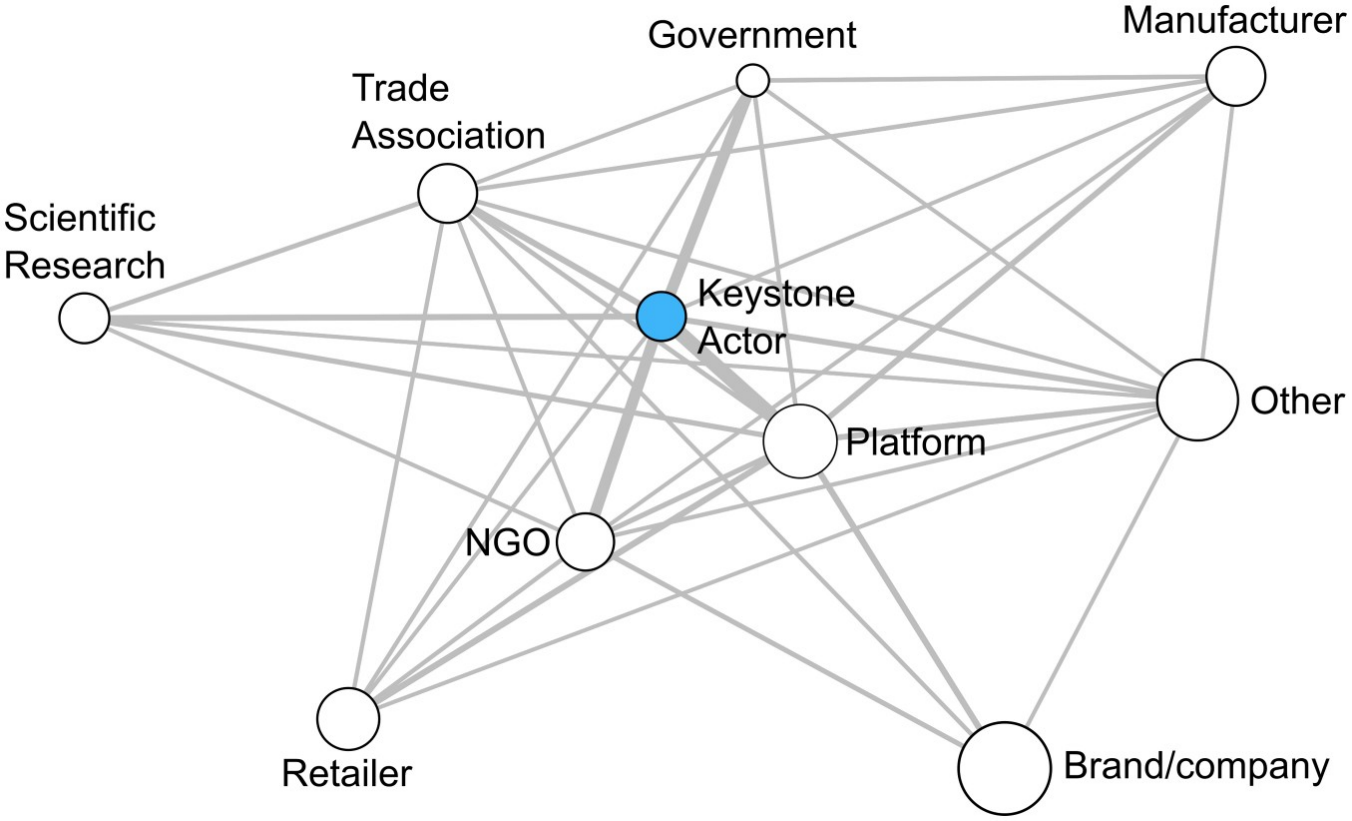
Visual representation of collaboration among different actor types in the business ecosystem. The frequency of collaboration is denoted by line thickness, while the size of the nodes corresponds to the number of actors belonging to each particular actor type.

The results of the E-I index analysis (Fig 3) illustrate how the business ecosystem is dominated by particular patterns of inter-organizational collaboration. External collaboration is more prevalent overall, and collaboration between keystone actors and multi-stakeholder platforms is the most frequent specific form. Other frequently occurring forms of external collaboration include: keystone actors and NGOs, multi-stakeholder platforms and retailers, keystone actors and trade associations, keystone actors and scientific research organizations, and keystone actors and government institutions. This last result is especially notable, given there are only six government institutions present in the business ecosystem. It suggests that while those government institutions in the ecosystem do tend to engage with keystone actors to address sustainability challenges in the global clothing industry, the system is largely governed through other regulatory processes, such as certification programs or audits.

The E-I index analysis also highlights gaps in collaboration in the business ecosystem. With the exception of collaboration among trade associations, internal collaboration is overall much less common than collaboration between different actor types. There is no direct collaboration among keystone actors, for example, although this result is not unexpected given the keystone actors are competitors. The same is also true for internal collaboration among clothing companies, retailers, and manufacturers, and for external collaboration between keystone actors and other clothing companies. These results broadly highlight the importance of collaboration for transforming unsustainable practices in global business ecosystems; keystone actors are competitors, and coordinating global action to achieve industry-wide sustainability is largely dependent on NGOs, multi-stakeholder platforms, and other organizations brokering indirect collaboration among keystone actors.

**Fig 4:**
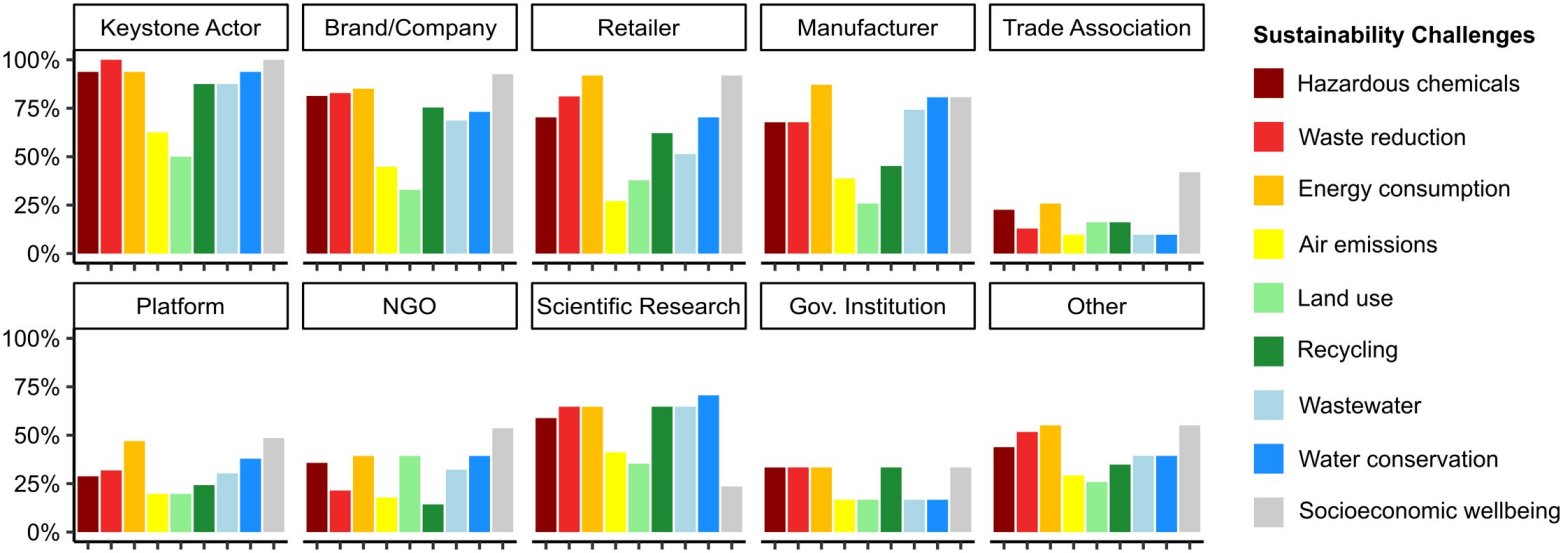
Biophysical and socioeconomic sustainability challenges addressed by different actor types. The number of challenges addressed is expressed as the fraction of the total actors belonging to each actor type that engage with each individual sustainability challenge.

In addition to structural contributions to the business ecosystem, there is also considerable variation across actor types in terms of the number and type of sustainability challenges they address (Table 3 and Fig 4). Keystone actors address a median of eight different challenges—the most of all actor types—while retailers and other clothing companies address a median of seven different challenges. The remaining actor types address considerably fewer sustainability challenges, which suggests these actors may specialize in subsets of challenges according to their areas of expertise. These results also serve to reinforce why collaboration is paramount for addressing industry-wide sustainability challenges; no single actor has all the requisite knowledge and resources to address complex biophysical and socioeconomic sustainability challenges spread across multiple sectors and countries throughout global supply chains.

Fig 4 illustrates the extent to which each type of actor in the business ecosystem engages with each of the nine biophysical and socioeconomic sustainability challenges. Socioeconomic issues represent the most frequently addressed sustainability challenge across nearly all actor types. The one exception is scientific research organizations, where socioeconomic issues rank last out of the nine challenges. Energy consumption is the most frequently addressed biophysical sustainability challenge overall, although there is variation across different types of actors. Aside from socioeconomic wellbeing and energy consumption, there is considerable variation across actor types in terms of the frequency with which water resource issues, hazardous chemicals, waste reduction, and recycling are addressed.

In spite of the fact the keystone actors are working to address most of the core biophysical and socioeconomic sustainability challenges, these efforts are not distributed equally across each individual issue. While efforts to address waste reduction and socioeconomic wellbeing were reported by all keystone actors, only around half of the keystone actors reported working to address air emissions and land use. These two issues are also among the least frequently addressed sustainability challenges overall, across all actor types. One exception is NGOs, where land use is the second most frequently addressed challenge, along with energy consumption. This may indicate that NGOs are important actors to consider when seeking collaborations to address land use issues in the global clothing industry.

## Discussion and conclusions

The results of the business ecosystem analysis have important implications for global governance processes, and highlight both gaps and opportunities for transforming unsustainable practices in the clothing industry. The overall open structure of the business ecosystem suggests it is currently structured around individual efforts to address sustainability challenges, as opposed to a coordinated industry-wide effort. On the one hand, it is important that keystone actors develop their own approaches for addressing the particularities of the sustainability challenges within their supply chains. These efforts, however, should not come at the expense of building industry-wide momentum for transformation, and should instead be leveraged to foster coordination more broadly among keystone actors [3]. On the other hand, studies have noted that the individualization of sustainability strategies can also signal a preference for voluntary programs and initiatives that deliver reputational benefits, but which may not result in improved social and ecological impacts [e.g., 66, 67].

While keystone actors have outsized impacts on global social-ecological systems, and have a critical role to play in sustainable transformations, the analysis here indicates they are by no means the only important actors to consider. The presence of a core group of central actors that span between the sixteen keystone actors in this study (Fig 2a)—primarily multi-stakeholder platforms and NGOs—may signal a potential leverage point for coordinating high-level action across the clothing industry. As other brands and clothing companies often look to keystone actors for guidance, and indeed often engage with many of the same multi-stakeholder platforms, sustainability strategies that focus on strengthening the mandates of these highly central actors may have positive spillover effects for the 134 other clothing companies in the ecosystem. However, to the extent that NGOs and multi-stakeholder sustainability platforms are dependent on government and/or corporate sponsorship, the central location of these actors in the business ecosystem may also indicate “network capture” by special interest groups [68].

Trade associations are one of the only actor types associated with closed network structures in the business ecosystem. Collaboration within dense communities of actors is important for facilitating collective-action to address complex, systemic sustainability challenges that cannot be resolved by any single actor alone. Yet trade associations in the global clothing industry are largely not focused on sustainability. In fact, sixteen of the thirty-one trade associations in this study do not report engaging with a single biophysical or socioeconomic sustainability challenge. Trade associations represent both a key gap and an unexploited opportunity; given their presence within dense communities of actors in the business ecosystem, it may be strategic to prioritize working with trade associations to adopt a sustainability focus. Furthermore, while the mission of trade associations has chiefly been to further the immediate economic interests of their members, many clothing companies recognize that, sooner or later, sustainability ultimately impacts their bottom lines.

The relative absence of government institutions in the business ecosystem is another important gap. Only six formal government actors are present, which further indicates the global clothing industry is largely governed by, and for, the industry. Without the involvement of regulatory authorities and accompanying legal mechanisms, efforts to implement sustainable practices taken by keystone actors and other clothing companies remain voluntary. The outcomes of these efforts will likely only be as good as the most stringent third-party certification programs that clothing companies voluntarily sign on to. Given biophysical and socioeconomic problems persist in the clothing industry, and indeed the impacts continue to grow, industry-led governance mechanisms alone do not appear sufficient for fostering wholesale transformation of unsustainable practices at the global scale.

Land use and air emissions tend to be the least frequently addressed sustainability challenges in the business ecosystem. This may signify a gap in global sustainability efforts, or it may mean that actors broadly perceive these particular challenges are being addressed. In the case of air emissions, reducing pollution is typically accomplished through technological and engineering solutions implemented at the factory level. It may also be that this issue is more commonly addressed at the country level. On the other hand, land use issues typically require companies to work at the front end of long supply chains, and to collaborate with a range of local and international organizations to address on-site production practices in different regions and countries. While we do not adopt a supply chain perspective here, teasing out these specifications is an important consideration for future studies.

Future research should also expand on the analysis we outline in other ways. Firstly, we focus on the process of collaboration to address sustainability challenges, not on actual performance measures. Therefore, the results of the business ecosystem analysis should be interpreted as the potential for addressing sustainability challenges. However, investigating outcomes requires companies to disclose considerable amounts of data that they currently do not make publicly available. Case studies of specific companies, or specific countries or regions, would allow for more in-depth analysis of social and environmental outcomes, especially where independent sustainability evaluations for global issues other than climate change become available. Secondly, future empirical research of the global clothing industry should also consider expanding the data collection process to include other brands and companies in addition to keystone actors. While we focus solely on the keystone actors here, it is likely that the data collection process still identifies the most important global organizations working to address unsustainable practices within the clothing industry. It is also telling that even though the business ecosystem was built around the keystone actors, they are not actually the most central actors in the ecosystem. Lastly, we do not consider the role of consumers here. It is important to recognize that companies exist because they have a customer base, and future research should consider addressing consumer behavior. Stated consumer preferences for sustainable clothing brands do not always translate to consumer purchasing behavior when such product lines are available [69].

Perhaps the biggest obstacle to overcome in applied research on keystone actors and global business ecosystems is one of agency. It is relatively easy to assess how different sustainability challenges are being addressed by different types of actors in an industry, but considerably more difficult to translate empirical research findings into actionable governance strategies. Specifically, who should be acting, and how? Folke et al. [1] begin to address these questions in their discussion of corporate biosphere stewardship and transformations toward sustainability, and provide a number of examples where actions are already occurring. By focusing on the ways in which keystone actors collaborate to address sustainability challenges, the conceptual and analytical approach we develop provides a means for beginning to operationalize such transformations. The approach we outline further highlights how industry-wide transformation requires recognizing the importance of other potentially influential organizations in the larger business ecosystems in which keystone actors are embedded. If the current high levels of industry attention to these issues continue into the future, then studies of the evolving structure of business ecosystems over time, coupled with social and environmental performance indicators, can help to identify particular strategies and actors that have been successful in advancing industry-wide transformation towards sustainability.
